# Direct search for dark matter axions excluding ALP cogenesis in the 63- to 67-μeV range with the ORGAN experiment

**DOI:** 10.1126/sciadv.abq3765

**Published:** 2022-07-06

**Authors:** Aaron Quiskamp, Ben T. McAllister, Paul Altin, Eugene N. Ivanov, Maxim Goryachev, Michael E. Tobar

**Affiliations:** 1 ARC Centre of Excellence for Engineered Quantum Systems and ARC Centre of Excellence For Dark Matter Particle Physics, Department of Physics, University of Western Australia, 35 Stirling Highway, Crawley, WA 6009, Australia.; 2 ARC Centre of Excellence for Dark Matter Particle Physics, Swinburne University of Technology, John St., Hawthorn, VIC 3122, Australia.; 3 ARC Centre of Excellence for Engineered Quantum Systems, The Australian National University, Canberra, ACT 2600, Australia.

## Abstract

The standard model axion seesaw Higgs portal inflation (SMASH) model is a well-motivated, self-contained description of particle physics that predicts axion dark matter particles to exist within the mass range of 50 to 200 micro–electron volts. Scanning these masses requires an axion haloscope to operate under a constant magnetic field between 12 and 48 gigahertz. The ORGAN (Oscillating Resonant Group AxioN) experiment (in Perth, Australia) is a microwave cavity axion haloscope that aims to search the majority of the mass range predicted by the SMASH model. Our initial phase 1a scan sets an upper limit on the coupling of axions to two photons of ∣*g*_aγγ_∣ ≤ 3 × 10^−12^ per giga–electron volts over the mass range of 63.2 to 67.1 micro–electron volts with 95% confidence interval. This highly sensitive result is sufficient to exclude the well-motivated axion-like particle cogenesis model for dark matter in the searched region.

## Teaser

The results of an axion dark matter search exclude the well-motivated axion-like particle cogenesis model for some masses.

## INTRODUCTION

The nature of dark matter remains one of the major unsolved problems in physics and astronomy with precise cosmological measurements, indicating that it accounts for 85% of all the matter in the universe (*1*, *2*). In this work, we undertake a direct search for one of the prime candidates, the dark matter axion, in a well-motivated mass range (*3*, *4*).

Axions are hypothetical, massive spin-0 particles that were first postulated as a result of an elegant solution to the strong charge-parity (CP) problem in quantum chromodynamics (QCD) (*5*–*7*). The weakly interacting nature of axions, combined with theoretical predictions and early-universe production mechanisms before or after inflation (*8*–*22*), simultaneously makes them a compelling dark matter candidate. To directly detect the dark matter axion, we may use a resonant cavity haloscope, which exploits the predicted axion two-photon coupling (*23*, *24*). Under this coupling, axions can convert to photons by interaction with another photon via the inverse Primakoff effect. In a typical haloscope experiment, a strong DC magnetic field is used to create a source of virtual photons for axions to interact with, producing real photons at a frequency corresponding to the rest mass of the axion, *m*_a_ (*23*, *24*), with some contribution from the axion velocity. In a typical haloscope, a resonant cavity with suitable mode geometry is used to capture these axion-converted photons, and the axion signal is read out with a low-noise amplification chain.

The received signal power in such an experiment, due to axion-photon conversion on resonance with the cavity, in the limit *Q*_L_ ≪ *Q*_a_ can be expressed as (*25*)

$$P_{\text{signal}}=(g_{\mathrm{a\gamma \gamma}}^{2}\frac{\rho_{a}}{m_{a}})(\frac{\beta }{1+\beta }B_{0}^{2}{\mathrm{VCQ}}_{L})$$ (1)

Here, the axion two-photon coupling strength *g*_aγγ_ [defined as per (*23*, *24*)], local dark matter density ρ_a_ ≈ 0.45 GeV/cm^3^ (assumed to be all axions), axion mass *m*_a_, and the expected signal quality factor *Q*_a_ ∼ 10^6^ [attributed to the axion kinetic energy distribution, most commonly cited as a Maxwell-Boltzmann distribution in the case of an isothermal, virialized halo (*26*, *27*)] are parameters set by nature. However, parameters controllable by the experimentalist include the magnetic field strength *B*_0_, cavity volume *V*, mode-dependent form factor *C*, and loaded quality factor *Q*_L_ = *Q*_0_/(1 + β), where *Q*_0_ and β denote the unloaded quality factor and receiver coupling strength, respectively. Another controllable parameter is the system noise temperature (*T*_S_) of the receiver, which represents the random Nyquist noise in the system in terms of the equivalent effective temperature. To minimize these fluctuations and to enable the use of a superconducting solenoidal magnet, a dilution refrigerator is typically used. Furthermore, since the axion mass and its coupling to photons are a priori unknown, experiments must tune (or scan) the haloscope resonant frequency, ν_c_, to match the axion frequency, ν_a_ ≈ *m*_a_*c*^2^/*h*.

The searchable value of the axion mass in theory may span many orders of magnitude; however, current models and astrophysical constraints suggest that the axion mass lies in the micro–electron volt–milli–electron volt region, for example, the standard model axion seesaw Higgs portal inflation (SMASH) model predicts 50 μeV ≤ *m*_a_ ≤ 200 μeV (*3*, *4*). In addition, recent QCD lattice simulations also favor 40 μeV ≤ *m*_a_ ≤ 180 μeV, with indications that the mass is close to 65 ± 6 μeV (14.2 to 17.2 GHz) (*28*), of which the mass range scanned in this experiment is a subset. However, since again the axion mass is unknown, experiments are required to span the largest possible axion mass range to maximize the prospects for discovery. Hence, the relevant figure of merit for axion haloscopes is the allowable rate of frequency scanning (*19*), given by (*25*)

$$\frac{\mathrm{df}}{\mathrm{dt}}=\frac{g_{a\gamma \gamma }^{4}}{{\text{SNR}}^{2}}\frac{\rho_{a}^{2}}{m_{a}^{2}}\frac{B_{0}^{4}V^{2}C^{2}}{k_{B}^{2}T_{S}^{2}}\frac{\beta^{2}}{{\left(1+\beta \right)}^{2}}\frac{Q_{L}Q_{a}^{2}}{Q_{L}+Q_{a}}$$ (2)

where *k*_B_ is the Boltzmann’s constant. The axion-photon coupling is parameterized by *g*_γ_, a dimensionless model-dependent number, taking a value of −0.97 and 0.36 in two benchmark QCD axion models, the Kim-Shifman-Vainshtein-Zakharov (KSVZ) and the Dine-Fischler-Srednicki-Zhitnitsky (DFSZ) models, respectively (*29*–*33*). So far, only a handful of experiments have reached KSVZ sensitivity (*34*–*37*), and only one experiment, the axion dark matter experiment, has reached the weaker DFSZ sensitivity (*38*–*40*).

Although most axion experiments target the QCD axion model bands, recent theoretical work suggests that more general ALP (axion-like particle) models are possible. An example is cogenesis, which predicts a much stronger axion-photon coupling as a consequence of adding ALPs to the standard model in the early universe to simultaneously explain the observed baryon and dark matter densities (*15*–*17*). Another example is the recent work on photophilic and photophobic axions, which shows that the parameter space for QCD axions may be much wider than conventional KSVZ and DFSZ axion models suggest (*20*, *41*).

**Fig. 1.**
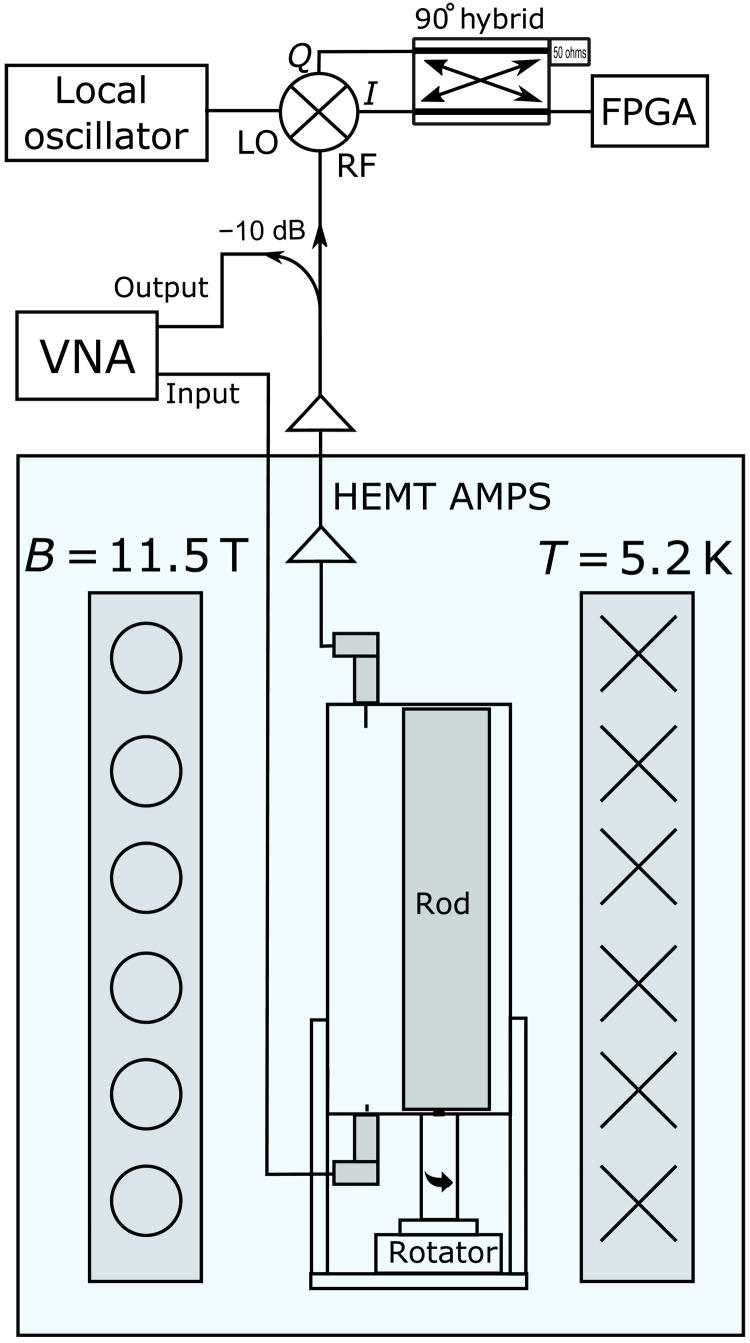
A diagram of the phase 1a experiment. A vector network analyzer (VNA) is used to measure the frequency response of the TM_010_ mode in transmission. An expected axion signal would be coupled out through the HEMT amplifier chain and mixed down using an IQ mixer and 90° hybrid coupler to be sampled by the field-programmable gate array (FPGA) digitizer. RF, radio frequency. AMPS, amplifiers.

The Oscillating Resonant Group AxioN (ORGAN) experiment is a microwave cavity haloscope hosted at the University of Western Australia, which aims to search for axions in the 62- to 207-μeV (15- to 50-GHz) mass region. The ORGAN run plan consists of several phases over different regions in the 15- to 50-GHz parameter space, with the first 26.5-GHz path-finding run already complete (*42*). We present the results of phase 1a (schematic shown in Fig. 1), which scans for axion masses in the 15.28- to 16.23-GHz (63- to 67-μeV) region of axion-photon coupling parameter space using a tunable TM_010_-based copper conducting rod resonator. This initial phase of ORGAN serves to test the ALP cogenesis model, operating at a physical temperature of ∼5.2 K in a 11.5-T magnetic field and using the best available low-noise high electron mobility transistor (HEMT) amplifiers. While this experiment is capable of placing sensitive limits on axion-photon coupling in its own right, it also serves as a path finder for future ORGAN phases, which aim to reach the QCD KSVZ and DFSZ model bands.

Compared to lower-frequency axion haloscopes, the disadvantage of the cavity resonator approach at the SMASH frequencies of interest is the necessarily small volume, which is of the order of the cube of the axion Compton wavelength, while the scan rate (Eq. 2) is proportional to the volume squared. This presents a substantial challenge for haloscopes going forward into the higher mass ranges. We are pursuing various parallel lines of research and development to mitigate this issue and achieve high sensitivity in the region of interest.

In the Supplementary Materials, we detail a path for the cavity resonator technique to be extended to reach KSVZ and DFSZ in the SMASH mass range. Subsequent stages of ORGAN will use the phase 1a infrastructure as a test bed for various technologies and techniques required to attain this sensitivity, such as gigahertz single-photon counting (*43*–*45*), cavity designs using dielectric-boosted sensitivity (*46*, *47*), and using multiple cavities simultaneously (*48*). Other experiments that target this mass range using the photon-axion chiral anomaly, such as the magnetized disc and Mirror axion experiment (MADMAX) (*49*) and the axion longitudinal plasma haloscope (ALPHA) (*50*), use other strategies to mitigate this problem, both are very promising, but currently in the research and development phase. Thus, it is still not experimentally clear which is the best technique to reach KSVZ and DFSZ sensitivity in this mass range. However, recently, an experiment reported limits on the product of electron and neutron pseudo-scalar coupling constants using a spin-based amplifier in a similar mass range (*51*).

## RESULTS

Using Eq. 1 and inserting typical values for our phase 1a detector, we find *P*_signal_ ≃ 2.3 × 10^−25^ (3.1 × 10^−26^) W for a 64-μeV KSVZ (DFSZ) axion. The total system noise referred to the receiver input is a function of the physical cavity temperature *T*_C_, the total added noise from the amplifier chain *T*_A_, and the detuning from resonance Δ = (ν − ν_c_)/Δν_c_, where Δν_c_ denotes the cavity linewidth. When the cavity and first amplifier are directly coupled with no isolation, *T*_S_ can be expressed as (*52*)

$$T_{S}=T_{C}\frac{4\beta }{{\left(1+\beta \right)}^{2}+4\Delta^{2}}+T_{A}\frac{1+4\Delta^{2}}{{\left(1+\beta \right)}^{2}+4\Delta^{2}}$$ (3)

For our parameters, the total system noise power is orders of magnitude greater than that from axion conversion at DFSZ sensitivity. However, the power generated by a cogenesis ALP is many orders of magnitude greater than the KSVZ or DFSZ axion, we calculate *P*_signal_ ≃ 8.5 × 10^−21^ W at 64 μeV, which is of similar order to the system noise in the ORGAN phase 1a experiment. To improve our ability to resolve any signal above these fluctuations, we must average many measurements to improve the signal-to-noise ratio (SNR). For a total integration time τ, the maximum achievable SNR is given by the Dicke radiometer (Eq. 4), where Δν_a_ is the expected axion linewidth

$$\text{SNR}=\frac{P_{\text{signal}}}{k_{B}T_{S}}\sqrt{\frac{\tau }{\Delta \nu_{a}}}$$ (4)

**Fig. 2.**
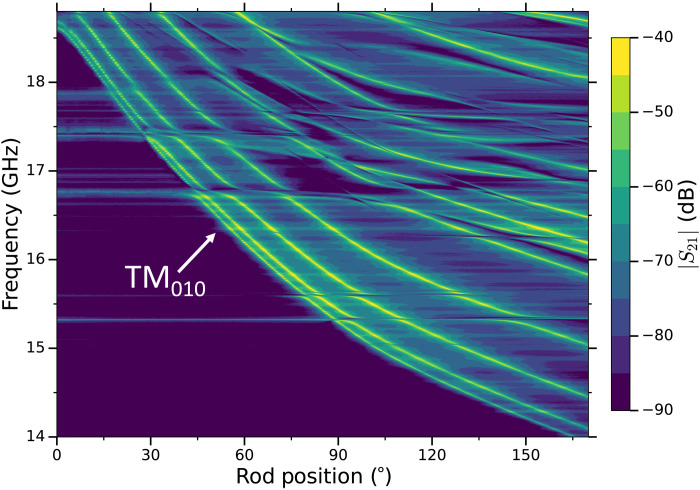
A color density plot of the transmission coefficient | *S*_21_ | (in decibels) as a function of resonant frequency and rod position. Lighter regions represent greater transmission, while darker regions represent lower transmission. The axion-sensitive TM_010_ mode is annotated as the lowest-order tunable mode. The horizontal lines are “intruding” transverse electric (TE) modes, which are discussed herein.

Before data taking began, we made a mode map of angular position versus frequency (see Fig. 2) to convert the required frequency step to stepper motor steps (see details in the Methods section). ORGAN phase 1a was run for ~2.5 weeks in September 2021 and a further week in January 2022, where typical parameters for the TM_010_ mode include, *Q*_L_ ≃ 3500, *C* ≃ 0.4, and β between 0.2 and 3. This initial phase was designed as the simplest implementation of the experiment with equipment on hand, the setup of which is shown in Fig. 1. The cavity is coupled directly to the amplifier to minimize losses, and instead of measuring the readout antenna coupling in situ during the experiment, we opted to set the coupling statically at room temperature and characterize it as a function of mode frequency, at cryogenic temperatures on separate, dedicated runs directly before and after the data-taking run. The average deviation in β between the “before data-taking” and “after data-taking” coupling characterization runs amounts to 10.1%, with an SD of 7.7%. This verifies that we have a good understanding of the coupling, without the need to measure it in situ. Our apparatus consists of a 32-mm–inner diameter copper cavity that houses a 16-mm-diameter copper tuning rod, which, at a cavity height of 80 mm, gives *V* ∼ 48 ml. The tuning rod was machined so that 0.2-mm gaps between the lid and the rod were present at either end of the rod, thus ensuring smooth tuning at cryogenic temperatures.

The coupling was set so that β ≃ 2 was reached at the operating temperature over as much of the target frequency range as possible. Since we had good knowledge of the coupling, with relative changes verified in situ by measuring the change in transmission spectrum, the integration time at each cavity step was varied between 45 and 210 min, depending on the value, so that cogenesis ALPs could be searched for or excluded over the entire accessible region.

During the total of ~3.5 weeks of data taking, 597 resonant frequencies were scanned, amounting to a search window of ~700 MHz between 15.28 and 16.23 GHz. Some of the data could not be used because of mode mixing between the axion-sensitive TM_010_ mode and intruding transverse electric (TE) modes, which arises as a result of the longitudinal symmetry breaking due to assembly tolerance in the axial position of the tuning rod (*53*). Mode interactions created forbidden frequency ranges between 15.58 and 15.61 GHz, 15.65 and 15.69 GHz , and 15.96 and 16.15 GHz, reducing the duty cycle of our search to ~74%. These regions will be probed in future, more sensitive iterations of the ORGAN experiment. At each cavity position, *Q*_L_, ν_c_, and Δν_c_ were extracted from transmission measurements, so that the local oscillator (LO) frequency could be set to ν_c_ − 45.1 MHz, thus placing the center of the mode at the center of the intermediate frequency (IF) window. Although the relatively high IF prevents unwanted spurious noise sources that are common at lower frequencies (IF ≤ 10 MHz), there were inescapable, large noise spikes at 40 and 50 MHz due to the device’s onboard 10-MHz oscillator, and so we restrict the analysis region to be between ~41.2 and 48.8 MHz.

We follow the The Haloscope At Yale Sensitive To Axion CDM (HAYSTAC) Experiment analysis procedure (*54*), which builds upon the pioneering work in (*55*). The first step is spectrum baseline removal, where the frequency-dependent baseline is a combination of the cavity thermal noise and the variable noise and gain of the readout chain. The broadband baseline of each spectrum can be removed using a Savitsky-Golay (SG) filter, which is a digital low-pass filter. The parameters of the SG filter must be chosen so that the pass-band is flat over large spectral scales, comparable to the baseline, while also maximizing stop band attenuation over smaller spectral scales, comparable to the axion signal width. As outlined in (*54*), the imperfect stop band attenuation of the SG filter induces small negative correlations between neighboring bins of the same spectrum and attenuates signals on small spectral scales, similar to an axion. This effect can be simulated and thus accounted for, but it requires knowledge of the full covariance matrix (see the Supplementary Materials).

Once filtered, the spectra are rescaled according to their axion sensitivity and then vertically combined using a maximum likelihood weighted sum of contributing spectra to maximize the SNR. As noted, the expected axion signal is thought to have *Q*_a_ ∼ 10^6^; hence, for ORGAN, a 15.6-GHz axion would have a linewidth of Δν_a_ ∼ 15.6 kHz ∼ 32Δν_b_. Therefore, we must horizontally combine bins from the vertically combined spectra so that the resulting “grand spectrum” bin width Δν_g_ is ∼Δν_a_. In addition, the expected Maxwell-Boltzmann axion line shape allows us to further enhance our sensitivity to potential axion signals by optimally filtering sets of 32 consecutive contributing bins according to this line shape.

**Fig. 3.**
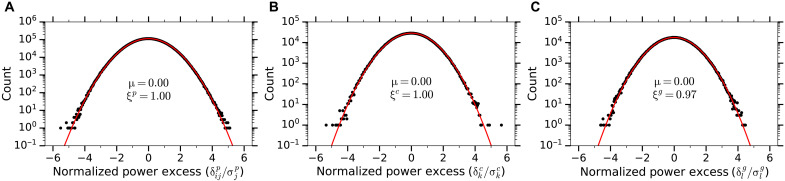
Histograms showing the normalized power spectra at different stages of the analysis procedure. Power excesses are shown as black circles, and the corresponding Gaussian fits are shown in red, where μ denotes the mean and ξ denotes the SD of the fit. (**A**) The distribution of the excess power spectra $\delta_{\mathrm{ij}}^{p}$ after being filtered by an SG fit and normalized to $\sigma_{j}^{p}$ is shown to be perfectly Gaussian. (**B**) Similarly, the vertical combination of processed spectra preserves the Gaussian nature of the noise, as shown by the distribution of normalized combined spectra $\delta_{k}^{c}/\sigma_{k}^{c}$. (**C**) The histogram of all grand spectrum bins $\delta_{k}^{g}/\sigma_{k}^{g}$ deviates from the standard normal Gaussian distribution by having a reduced width of ξ*^g^* = 0.97, which comes as a result of the negative SG filter–induced correlations between nearby bins that are coadded.

The excess power in the *k*th grand bin is denoted by $\delta_{k}^{g}$, and in the absence of correlations, the resulting normalized distribution $\delta_{k}^{g}/\sigma_{k}^{g}$ is expected to have a standard normal distribution. However, as shown in Fig. 3, the width of the grand spectrum distribution is reduced to ξ*^g^* = 0.97, which is a direct result of the negative correlations induced by the imperfect stop band attention of the SG filter. These negative correlations are accounted for through simulation (details are given in the Supplementary Materials), which allows us to place axion exclusion limits in a statistical manner, avoiding Monte Carlo simulations.

## DISCUSSION

**Fig. 4.**
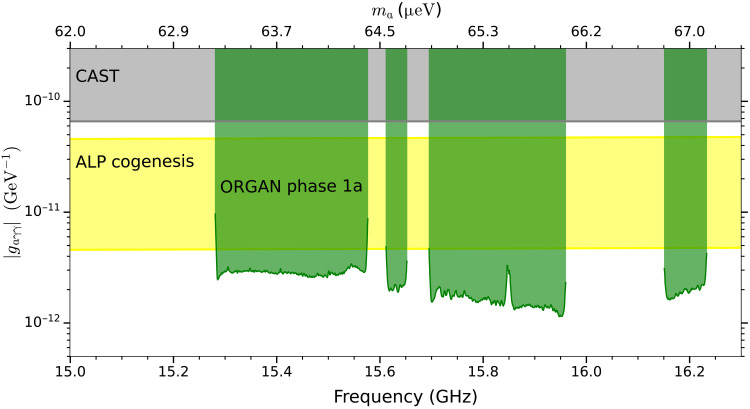
Our 95% confidence exclusion limits on the axion mass coupling parameter space. The results of this work are shown in green, surpassing the limits set by CAST (gray) (*57*) and beyond the ALP cogenesis model band (yellow) (*15*–*17*). The gaps in the exclusion plot correspond to mode-mixing regions where no axion-sensitive data could be taken. See the Supplementary Materials for a detailed analysis of the fractional uncertainty on these limits and future projections.

We place bin-by-bin exclusion limits on axion-photon coupling by assuming a constant target SNR and rescaling the sensitivity of each grand bin accordingly (*54*). To avoid rescans [which have been shown to have a negligible impact on sensitivity (*54*)], we set the candidate threshold at the maximum normalized power excess, 4.6σ, which corresponds to a target SNR of 6.245σ at 95% confidence interval. As shown in Fig. 4, we have surpassed the limit set by The CERN Axion Solar Telescope (CAST) by over an order of magnitude and excluded ALP cogenesis over most of the frequency range between 15.28 and 16.23 GHz (63 and 67 μeV), with the assumption that axions make up the total local dark matter density.

ORGAN is the first haloscope experiment to probe axions beyond 10.4 GHz (43 μeV) (*37*) into the Ku microwave band, and we have set the most sensitive limits to date on axion-photon coupling in this high-frequency region despite the unfavorable sensitivity scaling. We have detailed the operations and design of our first tunable cavity, with future plans to explore the entire 15- to 50-GHz parameter space at QCD model band coupling, with further details given in the Supplementary Materials. Development of quantum readout circuits required to reach this goal, such as gigahertz single-photon counters, is ongoing.

## METHODS

The readout chain, as shown in Fig. 1, consisted of a vector network analyzer (VNA) that measured (and later used) the frequency response of the TM_010_ mode in transmission, a cryogenic preamplifer, a directional coupler, and a down-conversion stage, which used a LO coupled to an in-phase (I) and quadrature (Q) mixer and 90° hybrid coupler. This down-mixing stage achieved an image rejection of the noisy, amplifier-only sideband, giving us the best possible *T*_S_. A 250 MS/s digitizer (NI-5761R) was used to sample the output of the hybrid coupler, and the digital data were processed in real time on a field-programmable gate array (FPGA; Xilinx Kintex-7, NI-7935R). A zero dead-time, hybrid superheterodyne–fast Fourier transform spectrum analyzer was implemented on the FPGA, which generated a 26,214 point, 12.5-MHz-wide spectrum centered at 45.1 MHz, with a bin width of Δν_b_ ≈ 477 Hz. Frequency tuning was achieved by rotating the off-axis rod with an Attocube ANR240 piezoelectric stepper motor, and steps between successive cavity positions were defined as a fraction of the cavity linewidth. A mode map of angular position versus frequency was made before data taking (see Fig. 2) and converted the required frequency step ∼Δν_c_/5 to stepper motor steps.

## Supplementary Materials

### This PDF file includes:

Sections S1 to S3 Figs. S1 to S3 Table S1 References
