# Supplementary material for: Direct search for dark matter axions excluding ALP cogenesis in the 63- to 67-μeV range with the ORGAN experiment
Source: Sci Adv. 2022 Jul 6;8(27):eabq3765. doi: 10.1126/sciadv.abq3765 (PMC9258816; doi:10.1126/sciadv.abq3765)
Supplement: Supplementary file 1 — Sections S1 to S3 Figs. S1 to S3 Table S1 References [file sciadv.abq3765_sm.pdf]

Science Advances
AAAS

Supplementary Materials for

Direct search for dark matter axions excluding ALP cogenesis in the 63- to 67-μeV range with the ORGAN experiment

Aaron Quiskamp *et al.*

Corresponding author: Aaron Quiskamp, aaron.quiskamp@research.uwa.edu.au; Ben T. McAllister, ben.mcallister@uwa.edu.au; Michael E. Tobar, michael.tobar@uwa.edu.au

Sci. Adv. **8**, eabq3765 (2022)
DOI: 10.1126/sciadv.abq3765

This PDF file includes:

Sections S1 to S3
Figs. S1 to S3
Table S1
References

S1: ORGAN Run Plan

The run detailed in the main body of the paper constitutes the ORGAN Phase 1a experiment. On the longer term, the ORGAN run plan consists of two discrete phases, as outlined in Figure S1.

Broadly speaking, Phase 1 consists of targeted searches utilizing readily available equipment and infrastructure, such as HEMT-based amplifiers, and tuning rod based resonators. Phase 1 searches will also serve as a testbed for various techniques and technologies to be implemented in future ORGAN searches. Phase 2 consists of more cutting edge searches, utilizing quantum technology, multiple cavity synchronization, novel resonators, and advanced readout techniques to enhance sensitivity. This is a broad characterization, as some of these features may be implemented from Phase 1b, pending research and development.

Phase 1, comprising Phases 1a and 1b, commenced in 2021 with Phase 1a. Phase 1b will operate on some of the same principles as Phase 1a (HEMT amplification, single cavity), but with key differences. Particularly, we intend to run Phase 1b at mK temperatures, rather than 4 K, to implement adjustable resonator coupling, and to utilize a novel dielectric resonant structure to enhance sensitivity [46, 47]. Phase 1b will cover $26.1 - 27.1$ GHz, to test the range of axion masses proposed by the Beck result [58, 59] and is projected to commence in 2022, running for a few months.

During Phase 1, we will continue research and development for future scale-ups, including the synchronization of multiple cavities - perhaps utilizing cross-correlation techniques [60], superconducting coatings and 3D printed structures [61] further novel resonant design, and single photon counters (SPCs) in the GHz frequency range [62, 63]. Some or all of these techniques will be implemented in Phase 2, pending the outcomes of the research and development.

If efficient GHz SPCs are developed within the timeline of Phase 1, it is possible that quick re-scans of the 1a and 1b regions will be undertaken with these devices, to develop expertise in their integration within a haloscope before Phase 2, and to extend the sensitivity of limits in the region. Efficient GHz SPCs are seen as the key research and development goal for future ORGAN phases, as they will allow access to the QCD model bands, and are well known to be far superior to even quantum limited linear amplification in these regimes [43].

Phase 2 is planned to commence in 2023, and is broken down into 5 GHz sub-phases, consisting of ~ 6 months of scanning on average, per sub-phase. The sub-phases will begin at 15 GHz, and end at 50 GHz, scanning the range proposed by the SMASH model [3,4]. Phase 2 will employ single photon counters if available by the beginning of the run, or quantum limited linear amplifiers as a fall-back.

Research and development for Phase 2 stages will be undertaken on a rolling basis, where Phase 2a cavities and detectors will be developed during Phase 1, and later Phase 2 stages will be developed during earlier Phase 2 stages.

Phase 2 will likely employ dielectric or other novel resonator designs, and consist of multiple cavities combined at the higher frequency end. If, as flagged above, single photon counters are implemented in Phase 1, this will save scan time in Phase 2, as 2 GHz of the range will have already been covered at similar sensitivity.

As with Phase 1, the use of efficient single photon counters will afford access to DFSZ sensitivity over the entire range, within the planned timeline, allowing time for expected maintenance and unplanned stops. If more traditional quantum-limited linear amplification is employed, a lower level of sensitivity will be attained, and efforts will be made to employ sub-quantum limited amplification, for example via a squeezed state receiver.

For all future projections, a SNR of 5, axion density of 0.45 GeV/cc, magnetic field of 12 T, and physical cavity temperature of $\sim$40-50 mK are assumed, along with form factors, volumes and quality factors from models of novel cavities [46, 47], and assumptions of either quantum limited amplification, or photon counters with efficiencies on the order of 0.5 and dark count rates of 1000 seconds/photon.

* aaron.quiskamp@research.uwa.edu.au
† ben.mcallister@uwa.edu.au
‡ michael.tobar@uwa.edu.au

Efficient GHz single photon counters are a field of on-going development. So far, the two most promising candidate technologies as far as axion haloscopes are concerned seem to be current-biased Josephson junctions (CBJJs) and cavity-coupled qubits (CCQs). Prototype CBJJs for axion haloscopes are currently in development around the world, with some promising initial results [62]. The ORGAN Collaboration is also pursuing research and development along these lines, and has acquired sample CBJJs which are being tested for future integration. Achieving a low dark count rate with the desired efficiency is the primary challenge, and the secondary challenge is coupling the device to a haloscope which sits inside a strong magnetic field. Progress is being made on the first front [63], whereas the solution to the second issue is likely to take the form of a superconducting transmission line.

On the CCQ front, such devices have already been applied in other, similar kinds of dark matter detection experiments [64], and work is underway to determine the path forward to integrating them with axion haloscopes.

So, whilst single photon counter development is a challenge, there are a clear paths forward, and we have identified a fall-back route utilizing well-tested quantum-limited amplification technology should all of these efforts fail. It is possible that Phase 2 will commence utilizing established quantum-limited amplifier technology, and transition to single photon counters part way through the run, as they become available. In this way, the run plan is flexible, and we have identified the expected sensitivity for both cases. It should be noted that the fall-back quantum-limited exclusion limits presented here could be improved by implementation of squeezed state receivers, which have already been demonstrated in axion haloscopes [36].

As shown in Figure S1, ORGAN has the potential to scan the entire $15-50$ GHz range with high sensitivity, within the decade.

S2: Data Analysis

The goal of the analysis is to search for significant power excesses above the noise that are consistent with an axion signal. If no such signals are found, limits can placed based on the sensitivity of our detector. We follow the HAYSTAC analysis procedure, with some minor modifications [65].

We start by discarding all data files that were not suitable for analysis, for example resonant frequencies that had poor reflection measurement fits, resulting in poor estimates for antenna coupling were discarded. We also remove all single bin contamination present in the intermediate frequency (IF) spectrum, since after very long integration times, the noise floor of the digitzer starts to become visible, with many small spikes scattered throughout the spectrum. The noise floor of the digitizer is measured by terminating the input and averaging for several hours. The bins that contained notable peaks above baseline ($\sigma \geq 5$) in the resulting IF spectrum are removed from the real data and replaced with a random value based on the standard deviation and mean in the 40 surrounding bins. In total, 38 out of 15,967 bins in the cropped analysis band were removed and replaced by a random value, thus preserving the Gaussian noise statistics.

The frequency dependent baseline is removed by a Savitsky-Golay (SG) filter which is parametrised by an impulse window length of $W = 450$ bins and polynomial order $d = 3$. To obtain a set of dimensionless, normalized excess power spectra, we subtract 1 and in the absence of axion conversion power each bin may be regarded as a Gaussian random variable with mean $\mu = 0$ and standard deviation $\sigma^p = 1/\sqrt{\Delta\nu_b\tau}$, where τ represents the total integration time and $\Delta\nu_b$ is the bin width. The baseline removal procedure is shown in Figure S2, where the raw data has been cropped to the usable bandwidth, corrected for small IF spikes, and subsequently filtered using an SG fit. The now flat excess power is normalized to σ^p from which we obtain Figure S2b.

The excess power in the jth IF bin of the ith processed spectrum is denoted by δ^p_{ij}, which when normalized to the standard deviation of the corresponding processed spectrum σ^p, is shown to obey Gaussian statistics, with standard deviation $\xi^p = 1$ and $\mu = 0$. This result demonstrates that the baseline removal procedure preserves the Gaussian nature of the thermal noise that we are measuring. As the cavity steps through frequency space, there will be a collection of different IF bins from multiple scans that will contribute to the same radio frequency (RF) bin. We follow the general procedure for vertical combination of multiple bins from different scans, using maximum likelihood (ML) weights [55,65]. However, before combination, each bin in each spectrum is re-scaled according to their axion sensitivity. We denote the scaled excess power in the jth IF bin of the ith spectrum by δ^s_{ij}, which is defined as

$$\delta^s_{ij} = \frac{\delta^p_{ij} k_B T^S_{ij} \Delta\nu_b}{P^{\text{signal}}_{ij}}. \tag{S2.1}$$

Here T^S_{ij} is the total system noise temperature referred to the input of the pre-amplifier for a given bin and P^{signal}_{ij} is the expected axion signal power in that bin. The standard deviation is weighted in the same way to obtain a set of scaled, IF bin dependent standard deviations denoted by σ^s_{ij} [65]. The rescaled spectra are then vertically combined

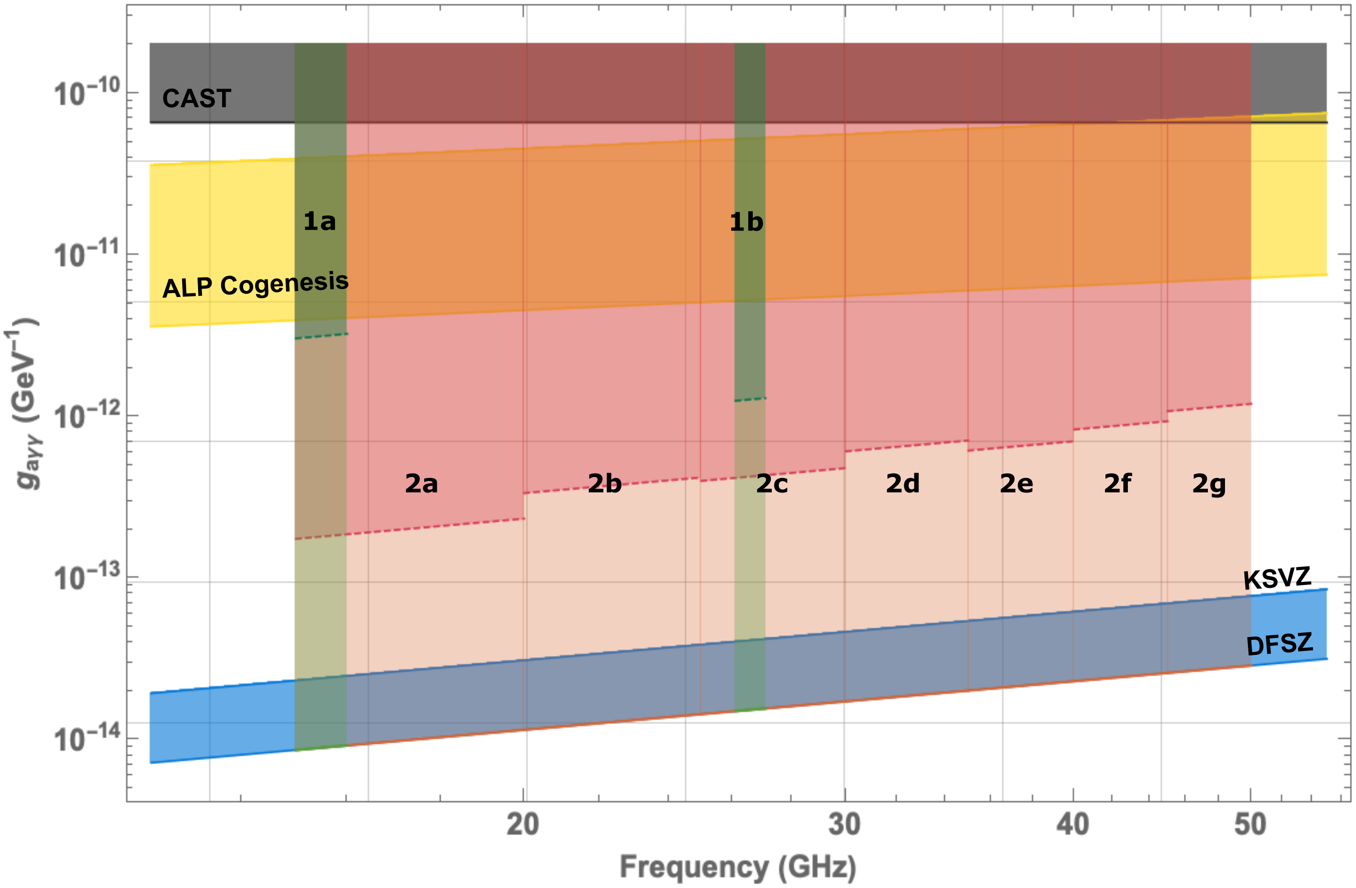

FIG. S1. **Projected exclusion limits based on different assumed parameters**. For Phase 1, the darker green limits represent what has already, been achieved or can be achieved using HEMT-based amplifiers, and resonators, with a form factor of 0.4, and a loaded Q of 10,000. As discussed, the current plan is to utilize a novel dielectric resonator for 1b - this may change pending research and development outcomes. The lighter green limits represent what can be achieved with single photon counters, should they be implemented in Phase 1. Phase 2 assumes a moderate scale up, utilizing dielectric resonators with a form factor of 0.4, a loaded quality factor of 30,000, and 50% greater volume than the Phase 1 resonators at the same frequency. The darker red limits represent what can be achieved with quantum limited linear amplifiers, whereas the lighter red limits represent what can be achieved with novel single photon counters with efficiencies on the order of 0.5, and dark count rates on the order of 1000 seconds/photon. The higher frequency limits assume combination of multiple $(2-4)$ cavities. Also shown are the common axion model bands [15-17, 29-33], and the exclusions from CAST [57].

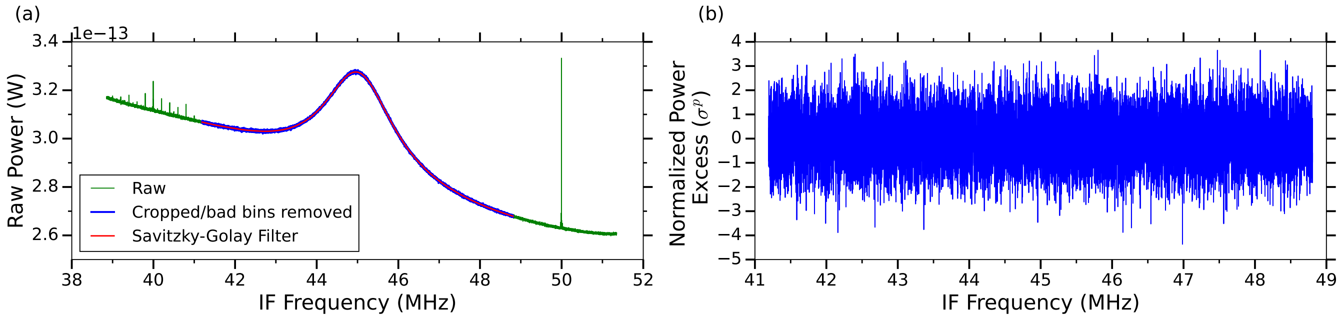

FIG. S2. **An example of the baseline removal procedure for a single cavity position**. (a) The raw spectrum (green) is cropped to the analysis region between $\sim 41.2-48.8$ MHz and all narrowband IF contamination is removed (blue). The resulting spectrum is then SG filtered (red). (b) We divide the cropped spectrum by the SG fit and subtracting 1 to give the processed spectrum, which is shown normalized to σ^p (blue).

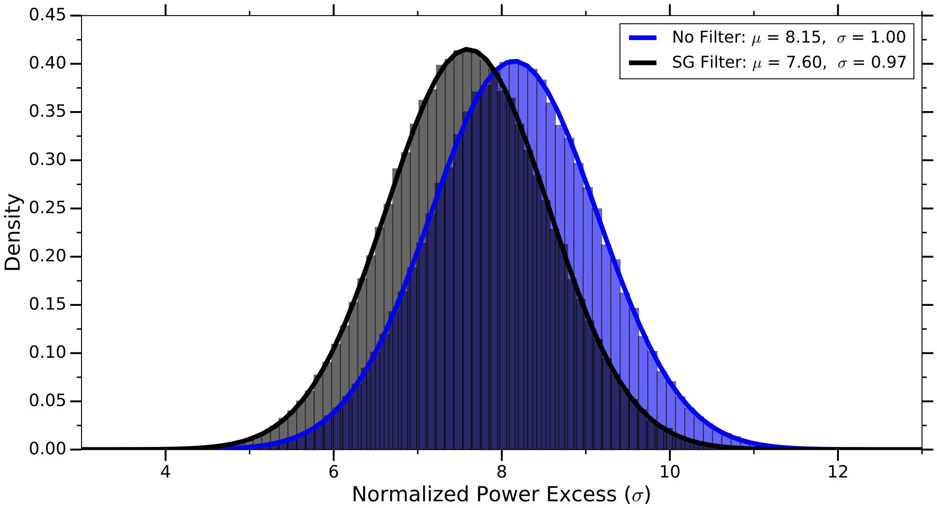

FIG. S3. **Histograms of the SNR for a synthetic axion signal injected into Gaussian white noise.** Here one dataset has been SG-filtered (black) and the other has not (blue). After 10^5 iterations of the simulation discussed in the text, we observe a standard normal distribution for the non-filtered data with $\sigma = 1$ and mean SNR $\mu = 8.15$, whereas the SG-filtered data results in a Gaussian distribution with reduced width $\sigma = 0.97$ and reduced mean SNR, $\mu = 7.60$. The negative correlations induced by the SG filter reduced the standard deviation by the same amount as that of the real data, with $\xi^g = 0.97$, further demonstrating our understanding of the origin of these correlations. The SG-filter induced attenuation of an axion signal can be expressed as the ratio of the two means $\eta\prime = 7.60/8.15 = 0.93$, which is then normalised to $\eta = \eta\prime/\xi^g = 0.96$ for reasons discussed in the text.

using a ML weighted sum of contributing spectra to maximize the SNR [55,65]. The resulting combined excess power in each RF bin is denoted by δ_k^c, where k represents the RF bin number. The combined standard deviation σ_k^c, for the kth bin is the quadrature weighted sum of contributing rescaled standard deviations. We expect the distribution of vertically combined bins, normalized to the expected power excess in each combined RF bin to be Gaussian, since independent scans are not correlated. This is shown to be the case, with a standard deviation of $\xi^c = 1$ and mean $\mu = 0$.

We enhance our sensitivity to axion detection by optimally filtering for the expected signal shape. In general, most haloscope searches assume a fully virialized local dark matter velocity distribution, with local circular velocity $v_0 = 220$ km/s. In the galactic frame, the spherically symmetric isothermal model obeys a Maxwell-Boltzmann distribution, and the resulting spectral shape is given by [65]

$$f(\nu) = \frac{2}{\sqrt{\pi}}\sqrt{\nu - \nu_a}\left(\frac{3}{\nu_a\left\langle\beta^2\right\rangle}\right)^{3/2} e^{-\frac{3(\nu - v_a)}{v_a\left\langle\rho^2\right\rangle}}, \tag{S2.2}$$

where ν and ν_a denote the measured and axion rest mass frequencies respectively and $\left\langle\beta^2\right\rangle = 3v_0^2/(2c^2)$, where c is the speed of light. The grand spectrum is constructed by horizontal combination of sets of 32 overlapping bin segments, which are ML-weighted sums that take into account the axion lineshape and the standard deviations σ_k^c, of contributing spectra. The standard deviation for each grand bin is the quadrature weighted sum of contributing standard deviations, which we denote as σ_k^g. As discussed in the main text, the width of the normalized distribution δ_k^g/σ_k^g is reduced to $\xi^g = 0.97$, as a result of the negative correlations induced by the imperfect stop-band attention of the SG filter. The degree of negative correlations between neighbouring bins is completely dependent on the choice of d and W, since these parameters decide the cutoff for the passband of the SG filter transfer function. We simulate the effect of the SG filter by comparing SG filtered and non-filtered Gaussian data. We compute the corrected standard deviation, which we denote by $\hat{\sigma}_k^g$, for a weighted sum of correlated Gaussian random variables [65]. In the case of negative filter-induced correlations, we would expect the sum of off diagonal elements of the covaraince matrix to be negative and hence $\hat{\sigma}_k^g$ will be reduced when compared to the un-corrected σ_k^g. We expect the ratio of $\hat{\sigma}_k^g/\sigma_k^g$ to be equal to the factor that the normalised grand spectrum width is reduced by, ξ^g. Through simulation we find this to be true, with $\hat{\sigma}_k^g/\sigma_k^g = \xi^g = 0.97$

The negative correlations imposed by the baseline removal procedure will also attenuate a potential axion signal. This reduction in SNR is also simulated, whereby a synthetic axion of known power is injected into 2 analysis pipelines

that contain Gaussian white noise. The first pipeline multiplies and then removes a randomly generated baseline using an SG filter, thus simulating the real data analysis procedure, whilst the second control pipeline does not include baseline removal. Once the grand spectrum has been constructed, the normalized power excess δ_k^g/σ_k^g, of the synthetic axion is recorded. The simulation is repeated over many iterations, and the results are shown as histograms in Figure S3. The reduction in SNR is represented by the ratio of means between the two pipelines, which we find to be $\eta\prime = 0.93$. Since the filter-induced negative correlations also reduce the random noise power fluctuations, as evidenced by the reduced width of the grand spectrum distribution, the total attenuation factor is also reduced by this same amount. We denote the total attenuation in SNR by $\eta = \eta\prime/\xi^g = 0.96$. The SNR of each grand bin to a given axion signal is then scaled by this amount to reflect the attenuated SNR, after which limits can be placed in a statistical manner, which are based on the sensitivity of our detector.

S3: Uncertainty Analysis

In this section we will quantify our best estimate for the fractional uncertainty in axion-photon coupling for the limits presented in the main text. Given a target SNR and confidence level, limits on $g_{a\gamma\gamma}$ will depend on the bin-by-bin grand spectrum SNR's, that are calculated using the expected axion power at the minimum excludable axion-photon coupling and the noise level of our detector at that frequency. Including only the parameters that carry the greatest uncertainty, we can express the minimum excludable axion-photon coupling $g_{a\gamma\gamma}^{min}$ in the following way,

$$g_{a\gamma\gamma}^{\rm min} \propto \sqrt{\frac{T_{\rm A}}{\frac{\beta}{1+\beta} C Q_L}}. \tag{S3.1}$$

We calculate the total added noise from the amplifier chain referred to the input of the pre-amplifier, $T_{\rm A}$, by using the calibrated frequency dependent effective noise temperature and gain provided by the manufacturer, Low Noise Factory. The fractional uncertainty associated with $T_{\rm A}$ is taken to be half the maximum range of $T_{\rm A}$ over the frequency region of interest. In future searches, we plan to calibrate this value more precisely *in situ*, thus reducing this uncertainty. As discussed in the main text, the antenna coupling β, was measured before and after the main data taking run. Therefore we estimate the fractional uncertainty as the mean deviation between these two runs, $\delta\beta/\beta \sim 10\%$. The loaded quality factor Q_L, is taken from the least squares fitting of transmission measurements to a Fano resonance model, which we find to give reliable and robust estimates of Q_L even in the presence of large asymmetry and noise [66]. Unlike other experiments which take the standard error from the least squares fit as the uncertainty, we opt for a more conservative approach by taking the average difference between the Fano fitted Q_L and the 3dB width estimate of Q_L. We model the frequency dependent form factor C for the TM_{010} mode of the Phase 1a cavity using Finite Element Method (FEM) modelling in COMSOL Multiphysics. Previous searches have taken the uncertainty in C to be $\sim 3-5\%$ [39, 40], from which we adopt the upper limit. Our estimates for the fractional uncertainties of the parameters in Eqn. S3.1 are tabulated below.

Source	Fractional Uncertainty
β	0.10
Q_L	0.18
C	0.05
T_A	0.08

TABLE S1. The dominant sources of systematic uncertainty which are discussed in the text.

We vary Eqn. S3.1 to find the total fractional uncertainty in $g_{a\gamma\gamma}^{min}$, and substitute the values in the Table S1 to arrive at Eqn. S3.2 as shown below, where $\bar{\beta}$ is the average coupling over the frequencies scanned. We estimate the total uncertainty in $g_{a\gamma\gamma}^{min}$ to be $\approx 10\%$.

$$\frac{\delta g_{a\gamma\gamma}^{min}}{g_{a\gamma\gamma}^{min}} \approx \sqrt{\left(\frac{1}{2}\frac{\delta T_{\rm A}}{T_{\rm A}}\right)^2 + \left(\frac{1}{2}\frac{\delta Q_L}{Q_L}\right)^2 + \left(\frac{1}{2}\frac{\delta C}{C}\right)^2 + \left(\frac{1}{2}\frac{1}{1+\bar{\beta}}\frac{\delta\beta}{\beta}\right)^2} \approx 10\% \tag{S3.2}$$

REFERENCES AND NOTES

1. Planck Collaboration, Planck 2015 results. XIII. Cosmological parameters. *Astron. Astrophys.* **594**, A13 (2016)

2. C. L. Bennett, D. Larson, J. L. Weiland, N. Jarosik, G. Hinshaw, N. Odegard, K. Smith, R. Hill, B. Gold, M. Halpern, E. Komatsu, M. R. Nolta, L. Page, D. N. Spergel, E. Wollack, J. Dunkley, A. Kogut, M. Limon, S. S. Meyer, G. S. Tucker, E. L. Wright, Nine-year Wilkinson Microwave Anisotropy Probe (WMAP) observations: Final maps and results. *Astrophys. J. Suppl. Ser.* **208**, 20 (2013).

3. G. Ballesteros, J. Redondo, A. Ringwald, C. Tamarit, Unifying inflation with the axion, dark matter, baryogenesis, and the seesaw mechanism. *Phys. Rev. Lett.* **118**, 071802 (2017).

4. G. Ballesteros, J. Redondo, A. Ringwald, C. Tamarit, Several problems in particle physics and cosmology solved in one smash. *Front. Astron. Space Sci.* **6**, 55 (2019).

5. R. D. Peccei, H. R. Quinn, Cp conservation in the presence of pseudoparticles. *Phys. Rev. Lett.* **38**, 1440–1443 (1977).

6. S. Weinberg, A new light boson? *Phys. Rev. Lett.* **40**, 223–226 (1978).

7. F. Wilczek, Problem of strong *p* and *t* invariance in the presence of instantons. *Phys. Rev. Lett.* **40**, 279–282 (1978).

8. L. Abbott, P. Sikivie, A cosmological bound on the invisible axion. *Phys. Lett. B* **120**, 133–136 (1983).

9. J. Ipser, P. Sikivie, Can galactic halos be made of axions? *Phys. Rev. Lett.* **50**, 925–927 (1983).

10. J. Preskill, M. B. Wise, F. Wilczek, Cosmology of the invisible axion. *Phys. Lett. B* **120**, 127–132 (1983).

11. P. Svrcek, E. Witten, Axions in string theory. *J. High Energy Phys.* **2006**, 051 (2006).

12. A. Arvanitaki, S. Dimopoulos, S. Dubovsky, N. Kaloper, J. March-Russell, String axiverse. *Phys. Rev. D* **81**, 123530 (2010)

13. T. Higaki, K. Nakayama, F. Takahashi, Cosmological constraints on axionic dark radiation from axion-photon conversion in the early universe. *J. Cosmol. Astropart. Phys.* **2013**, 030 (2013).

14. D. Baumann, D. Green, B. Wallisch, New target for cosmic axion searches. *Phys. Rev. Lett.* **117**, 171301 (2016).

15. R. T. Co, L. J. Hall, K. Harigaya, Axion kinetic misalignment mechanism. *Phys. Rev. Lett.* **124**, 251802 (2020).

16. R. T. Co, K. Harigaya, Axiogenesis. *Phys. Rev. Lett.* **124**, 111602 (2020).

17. R. T. Co, L. J. Hall, K. Harigaya, Predictions for axion couplings from alp cogenesis. *J. High Energy Phys.* **2021**, 172 (2021).

18. V. K. Oikonomou, Unifying inflation with early and late dark energy epochs in axion $f(r)$ gravity. *Phys. Rev. D* **103**, 044036 (2021).

19. P. Sikivie, Invisible axion search methods. *Rev. Mod. Phys.* **93**, 015004 (2021).

20. A. V. Sokolov, A. Ringwald, Photophilic hadronic axion from heavy magnetic monopoles. *J. High Energy Phys.* **2021**, 123 (2021).

21. L. Di Luzio, M. Giannotti, E. Nardi, L. Visinelli, The landscape of QCD axion models. *Phys. Rep.* **870**, 1–117 (2020).

22. J. A. Dror, H. Murayama, N. L. Rodd, Cosmic axion background. *Phys. Rev. D* **103**, 115004 (2021).

23. P. Sikivie, Experimental tests of the “invisible” axion. *Phys. Rev. Lett.* **51**, 1415–1417 (1983).

24. P. Sikivie, Experimental tests of the “invisible” axion. *Phys. Rev. Lett.* **52**, 695 (1984).

25. D. Kim, J. Jeong, S. Youn, Y. Kim, Y. K. Semertzidis, Revisiting the detection rate for axion haloscopes. *J. Cosmol. Astropart. Phys.* **2020**, 066 (2020).

26. M. S. Turner, Periodic signatures for the detection of cosmic axions. *Phys. Rev. D* **42**, 3572–3575 (1990).

27. R. Jimenez, L. Verde, S. P. Oh, Dark halo properties from rotation curves. *Mon. Not. R. Astron. Soc.* **339**, 243–259 (2003).

28. M. Buschmann, J. W. Foster, A. Hook, A. Peterson, D. E. Willcox, W. Zhang, B. R. Safdi, Dark matter from axion strings with adaptive mesh refinement. *Nat. Commun.* **13**, 1049 (2022).

29. J. E. Kim, Weak-interaction singlet and strong CP invariance. *Phys. Rev. Lett.* **43**, 103–107 (1979).

30. A. R. Zhitnitsky, On possible suppression of the axion hadron interactions. (In Russian). *Sov. J. Nucl. Phys.* **31**, 260 (1980)

31. M. Dine, W. Fischler, M. Srednicki, A simple solution to the strong {CP} problem with a harmless axion. *Phys. Lett. B* **104**, 199–202 (1981).

32. M. Shifman, A. Vainshtein, V. Zakharov, Can confinement ensure natural *CP* invariance of strong interactions? *Nucl. Phys. B* **166**, 493–506 (1980).

33. M. Dine, W. Fischler, The not-so-harmless axion. *Phys. Lett. B* **120**, 137–141 (1983).

34. O. Kwon, D. Lee, W. Chung, D. Ahn, H. Byun, F. Caspers, H. Choi, J. Choi, Y. Chong, H. Jeong, J. Jeong, J. E. Kim, J. Kim, Ç. Kutlu, J. Lee, M. J. Lee, S. Lee, A. Matlashov, S. Oh, S. Park, S. Uchaikin, S. W. Youn, Y. K. Semertzidis, First results from an axion haloscope at capp around 10:7 μeV. *Phys. Rev. Lett.* **126**, 191802 (2021).

35. S. Lee, S. Ahn, J. Choi, B. R. Ko, Y. K. Semertzidis, Axion dark matter search around 6.7 μev. *Phys. Rev. Lett.* **124**, 101802 (2020).

36. K. M. Backes, D. A. Palken, S. A. Kenany, B. M. Brubaker, S. B. Cahn, A. Droster, G. C. Hilton, S. Ghosh, H. Jackson, S. K. Lamoreaux, A. F. Leder, K. W. Lehnert, S. M. Lewis, M. Malnou, R. H. Maruyama, N. M. Rapidis, M. Simanovskaia, S. Singh, D. H. Speller, I. Urdinaran, L. R. Vale, E. C. van Assendelft, K. van Bibber, H. Wang, A quantum enhanced search for dark matter axions. *Nature* **590**, 238–242 (2021).

37. D. Alesini, C. Braggio, G. Carugno, N. Crescini, D. D'Agostino, D. Di Gioacchino, R. Di Vora, P. Falferi, U. Gambardella, C. Gatti, G. Iannone, C. Ligi, A. Lombardi, G. Maccarrone, A. Ortolan, R. Pengo, A. Rettaroli, G. Ruoso, L. Taffarello, S. Tocci, Search for invisible axion dark matter of mass m_a = 43 μeV with the quax–aγ experiment. *Phys. Rev. D* **103**, 102004 (2021).

38. T. Braine, R. Cervantes, N. Crisosto, N. Du, S. Kimes, L. J. Rosenberg, G. Rybka, J. Yang, D. Bowring, A. S. Chou, R. Khatiwada, A. Sonnenschein, W. Wester, G. Carosi, N. Woollett, L. D. Duffy, R. Bradley, C. Boutan, M. Jones, B. H. LaRoque, N. S. Oblath, M. S. Taubman, J. Clarke, A. Dove, A. Eddins, S. R. O'Kelley, S. Nawaz, I. Siddiqi, N. Stevenson, A. Agrawal, A. V. Dixit, J. R. Gleason, S. Jois, P. Sikivie, J. A. Solomon, N. S. Sullivan, D. B. Tanner, E. Lentz, E. J. Daw, J. H. Buckley, P. M. Harrington, E. A. Henriksen, K. W. Murch; ADMX Collaboration, Extended search for the invisible axion with the axion dark matter experiment. *Phys. Rev. Lett.* **124**, 101303 (2020).

39. C. Bartram, T. Braine, R. Cervantes, N. Crisosto, N. Du, G. Leum, L. J. Rosenberg, G. Rybka, J. Yang, D. Bowring, A. S. Chou, R. Khatiwada, A. Sonnenschein, W. Wester, G. Carosi, N. Woollett, L. D. Duffy, M. Goryachev, B. McAllister, M. E. Tobar, C. Boutan, M. Jones, B. H. LaRoque, N. S. Oblath, M. S. Taubman, J. Clarke, A. Dove, A. Eddins, S. R. O'Kelley, S. Nawaz, I. Siddiqi, N. Stevenson, A. Agrawal, A. V. Dixit, J. R. Gleason, S. Jois, P. Sikivie, J. A. Solomon, N. S. Sullivan, D. B. Tanner, E. Lentz, E. J. Daw, M. G. Perry, J. H. Buckley, P. M. Harrington, E. A. Henriksen; K. W. Murch; ADMX Collaboration, Axion dark matter experiment: Run 1B analysis details. *Phys. Rev. D* **103**, 032002 (2021).

40. C. Bartram, T. Braine, E. Burns, R. Cervantes, N. Crisosto, N. Du, H. Korandla, G. Leum, P. Mohapatra, T. Nitta, L. J. Rosenberg, G. Rybka, J. Yang, J. Clarke, I. Siddiqi, A. Agrawal, A. V. Dixit, M. H. Awida, A. S. Chou, M. Hollister, S. Knirck, A. Sonnenschein, W. Wester, J. R.

Gleason, A. T. Hipp, S. Jois, P. Sikivie, N. S. Sullivan, D. B. Tanner, E. Lentz, R. Khatiwada, G. Carosi, N. Robertson, N. Woollett, L. D. Duffy, C. Boutan, M. Jones, B. H. LaRoque, N. S. Oblath, M. S. Taubman, E. J. Daw, M. G. Perry, J. H. Buckley, C. Gaikwad, J. Hoffman, K. W. Murch, M. Goryachev, B. T. McAllister, A. Quiskamp, C. Thomson, M. E. Tobar; ADMX Collaboration, Search for invisible axion dark matter in the 3.3-4.2 μeV mass range. *Phys. Rev. Lett.* **127**, 261803 (2021).

41. N. Craig, A. Hook, S. Kasko, The photophobic alp. *J. High Energy Phys.* **2018**, 28 (2018).

42. B. T. McAllister, G. Flower, E. N. Ivanov, M. Goryachev, J. Bourhill, M. E. Tobar, The organ experiment: An axion haloscope above 15 GHz. *Phys. Dark Universe* **18**, 67–72 (2017).

43. S. K. Lamoreaux, K. A. van Bibber, K. W. Lehnert, G. Carosi, Analysis of single-photon and linear amplifier detectors for microwave cavity dark matter axion searches. *Phys. Rev. D* **88**, 035020 (2013).

44. L. S. Kuzmin, A. S. Sobolev, C. Gatti, D. Di Gioacchino, N. Crescini, A. Gordeeva, E. Il'ichev, Single photon counter based on a Josephson junction at 14 GHz for searching galactic axions. *IEEE Trans. Appl. Supercond.* **28**, (2018).

45. A. Rettaroli, D. Alesini, D. Babusci, C. Barone, B. Buonomo, M. M. Beretta, G. Castellano, F. Chiarello, D. Di Gioacchino, G. Felici, G. Filatrella, L. G. Foggetta, A. Gallo, C. Gatti, C. Ligi, G. Maccarrone, F. Mattioli, S. Pagano, S. Tocci, G. Torrioli, Josephson junctions as single microwave photon counters: Simulation and characterization. *Instruments* **5**, 25 (2021).

46. B. T. McAllister, G. Flower, L. E. Tobar, M. E. Tobar, Tunable supermode dielectric resonators for axion dark-matter haloscopes. *Phys. Rev. Appl.* **9**, 014028 (2018).

47. A. P. Quiskamp, B. T. Mcallister, G. Rybka, M. E. Tobar, Dielectric-boosted sensitivity to cylindrical azimuthally varying transverse-magnetic resonant modes in an axion haloscope. *Phys. Rev. Appl.* **14**, 044051 (2020).

48. B. T. Mcallister, S. R. Parker, E. N. Ivanov, M. E. Tobar, Cross-correlation signal processing for axion and wisp dark matter searches. *IEEE Trans. Ultrason. Ferroelectr. Freq. Control* **66**, 236–243 (2019).

49. A. Caldwell, G. Dvali, B. Majorovits, A. Millar, G. Raffelt, J. Redondo, O. Reimann, F. Simon, F. Steffen; MADMAX Working Group, Dielectric haloscopes: A new way to detect axion dark matter. *Phys. Rev. Lett.* **118**, 091801 (2017).

50. M. Lawson, A. J. Millar, M. Pancaldi, E. Vitagliano, F. Wilczek, Tunable axion plasma haloscopes. *Phys. Rev. Lett.* **123**, 141802 (2019).

51. Y. Wang, H. Su, M. Jiang, Y. Huan, Y. Qin, C. Guo, Z. Wang, D. Hu, W. Ji, P. Fadeev, X. Peng, D. Budker, Limits on axions and axionlike particles within the axion window using a spin-based amplifier. arXiv:2201.11847 [physics.ins-det] (24 January 2022).

52. M. Xu, X. Han, C.-L. Zou, W. Fu, Y. Xu, C. Zhong, L. Jiang, H. X. Tang, Radiative cooling of a superconducting resonator. *Phys. Rev. Lett.* **124**, 033602 (2020).

53. I. Stern, G. Carosi, N. Sullivan, D. Tanner, Avoided mode crossings in cylindrical microwave cavities. *Phys. Rev. Appl.* **12**, 044016 (2019).

54. B. M. Brubaker, L. Zhong, Y. V. Gurevich, S. B. Cahn, S. K. Lamoreaux, M. Simanovskaia, J. R. Root, S. M. Lewis, S. A. Kenany, K. M. Backes, I. Urdinaran, N. M. Rapidis, T. M. Shokair, K. A. V. Bibber, D. A. Palken, M. Malnou, W. F. Kindel, M. A. Anil, K. W. Lehnert, G. Carosi, First results from a microwave cavity axion search at 24 μeV. *Phys. Rev. Lett.* **118**, 061302 (2017).

55. S. Asztalos, E. Daw, H. Peng, L. J. Rosenberg, C. Hagmann, D. Kinion, W. Stoeffl, K. van Bibber, P. Sikivie, N. S. Sullivan, D. B. Tanner, F. Nezrick, M. S. Turner, D. M. Moltz, J. Powell, M.-O. André, J. Clarke, M. Mück, R. F. Bradley, Large-scale microwave cavity search for dark-matter axions. *Phys. Rev. D* **64**, 092003 (2001).

56. A. Quiskamp, B. McAllister, M. Tobar, Direct Search for Dark Matter Axions Excluding ALP Cogenesis in the 63–67 micro-eV Range, with the ORGAN experiment (2022); https://zenodo.org/record/6539102.

57. CAST Collaboration, New cast limit on the axion-photon interaction. *Nat. Phys.* **13**, 584–590 (2017).

58. C. Beck, Possible resonance effect of axionic dark matter in josephson junctions. *Phys. Rev. Lett.* **111**, 231801 (2013).

59. C. Beck, Axion mass estimates from resonant josephson junctions. *Phys. Dark Universe* **7-8**, 6 (2015).

60. B. T. McAllister, S. R. Parker, E. N. Ivanov, M. E. Tobar, Cross-correlation signal processing for axion and wisp dark matter searches. *IEEE Trans. Ultrason. Ferroelectr. Freq. Control* **66**, 236 (2018).

61. D. Ahn, D. Youm, O. Kwon, W. Chung, Y. K. Semertzidis, High quality factor high-temperature superconducting microwave cavity development for the dark matter axion search in a strong magnetic field. arXiv preprint arXiv:1902.04551 (2019).

62. L.S. Kuzmin, A. S. Sobolev, C. Gatti, D. Di Giocchino, N. Crescini, A. Gordeeva, E. Il'ichev, Single photon counter based on a josephson junction at 14 ghz for searching galactic axions. *IEEE Trans. Appl. Supercond.* **28**, 1 (2018).

63. D. S. Golubev, E. V. Il'ichev, L. S. Kuzmin, Single-photon detection with a josephson junction coupled to a resonator. *Phys. Rev. Appl.* **16**, 014025 (2021).

64. A. V. Dixit, S. Chakram, K. He, A. Agrawal, R. K. Naik, D. I. Schuster, A. Chou, Searching for dark matter with a supeconducting qubit. *Phys. Rev. Lett.* **126**, 141302 (2021).

65. B. M. Brubaker, L. Zhong, S. K. Lamoreaux, K. W. Lehnert, K. A. V. Bibber, Haystac axion search analysis procedure. *Phys. Rev. D* **96**, 10.1103/PhysRevD.96.123008 (2017).

66. U. Fano, Effects of configuration interaction of intensities and phase shift. *Phys. Rev.* **124**, 1866 (1961).
